# Management in Indian blood banking system: True reality

**DOI:** 10.4103/0973-6247.53871

**Published:** 2009-07

**Authors:** N. Choudhury

**Affiliations:** *Coordinator, Blood Safety; Gujarat State Council for Blood Transfusion, O-1 New MH Complex, Meghaninagar, Ahmedabad-380016, India*

Blood transfusion service is a multibillion dollar profession/ business worldwide. Being Indian blood bank personnel, we may not like to call it as business. But in the real sense, it is a production industry with all the components of business built into it. Still, we will not like to call it a business because of the fear in our minds that our general population or blood donors will be annoyed with us and may not come to donate again. Many a times, we do not clarify to the donor at the time of donation that there is service charge for blood units. As a result, when the donor needs blood, it comes as a rude shock and he makes allegations of the ‘sale’ of blood. At that moment, the situation becomes embarrassing because we hesitate to accept that we follow a cost recovery system which is a part of the business.

Many of our friends may not like to call it a business. But do you ever think about the money involved in Indian blood transfusion services (BTS)? Let us calculate the figure. If we take the total blood collection in India as 7.5 million units yearly, 2% of blood is discarded (minimum) due to various reasons. If we deduct 2% of discarded blood, the total usable whole blood or red cells will be 6460,000 units in India. For blood components, let us take a conservative estimate that only 25% blood is separated into components. In that situation, we will have about 1,365,000 components for patients. Now to find out the total revenue generation across the country, let us take the service charge ceiling laid down by the National AIDS Control Organization (NACO). NACO has prescribed Rs. 850 per unit of whole blood or RBC and 6460,000 units will generate Rs.549,1000,000. On the other hand, components will attract revenue of Rs.68,2500,000 (@ Rs.500 per component on an average). Total revenue generated by whole blood/ red cells and components is Rs.617,3500,000 (or US$123270000 @ 1 USD= Rs.50). We have not calculated products prepared by aphaeresis technique because of non-availability of data. What does all this calculation mean to us? Indian BTS is a billion-dollar (Rs.617 crore) healthcare system and all of us are contributors to it.

Who are the managers of the Indian BTS, of this billion-dollar healthcare system? We have four types of blood banks/centers (from the administrative point of view) in India. They are managed by the public (government) sector, Indian Red Cross Society (IRCS), non-government organizations (NGOs, on not for profit basis) and corporate or commercial sectors. Let us discuss today how efficiently more than 2,460 blood banks in India are managed. Roughly, about 55% blood banks are from the government sector, 5% from the IRCS, about 20-25% are from the NGO sector and the rest are from corporate or profit-making sectors. If we look into the government sector, blood banks are run by blood bank in-charge or Blood Transfusion Officers (BTOs) who takes care of regular administration under the guidance (?) of the Medical Superintendents/ Director of that hospital. Many a times, the head of laboratory or pathology also takes charge of the blood bank. If it is a medical college (government or private), usually the head of the department of Pathology takes additional responsibilities of the blood bank. There are about 40 blood banks in the country which are separated from the Pathology department as independent Transfusion Medicine (Immunohematology & Blood Transfusion) department where trained technical personnel take care of regular administration. In case of IRCS blood banks, there is a committee which ‘officially’ manages administration through the blood bank in-charge or BTO. However, there is always some control from the local or state-level IRCS in the management. In the fourth sector, i.e. in the corporate sector, management is in better hands. Because they have to manage mainly with a small number of family replacement donations, professional hospital administrators or experienced businessmen manage blood banks behind the scene.

Our point of discussion in this issue is the management of blood banks in the NGO or in the private sector. Why do some NGOs want to start blood banks? There are various reasons. The most common reason is philanthropy. If an NGO wants to do philanthropic work in the medical field, usually they want to start a blood bank. Probably, blood bank runs on philanthropic voluntary blood donation and these NGOs want to supply blood at a cheaper price. They initially fail to realize that it is a branch of medicine which needs sophisticated equipment and highly skilled manpower. Many non-medical trustees or promoters do not realize that supply of safe blood is more important than giving free blood. When the nitty-gritty of the Drugs and Cosmetics Act comes, they fall back on a few known medical personnel for help. In some stages trustees come to realize the hard fact that blood banking is not as simple as managing their own business/ profession. Then, they want some qualified medical personnel who can run the blood bank. But the issue is that they do want to keep control on every aspect of the blood bank, from blood collection to issue. The medical officer in this type of situation becomes confused and feels obstructed in his work. Many blood bank medical officers across the country feel that they are not allowed to work with a free hand and frequently face obstruction even in technical work. Of course, this is being felt in the corporate sector also. This leads to frustration and points of differences between Trustees and the technical head of the blood bank. However, both sides try to compromise and the work goes on, may not be as smoothly as before. In other situations, when it goes beyond compromise, one has to leave. Who leaves? Trustees will not leave because it is their fiefdom till their last breath. Therefore, the medical officer goes for another option.

Why are NGO Trustees motivated to start blood banks, in many cases, when they are not from the medical profession? On the face of it, it is philanthropy. Many people feel that it is not the only reason. It is widely felt that they want to become a part of the ‘business’ in multi-billon rupees and to get a share in it. Becoming a Trustee in any blood bank gives you name and fame in the medical profession. ‘I can arrange blood during emergency from my blood bank’. This message gives personal mileage in the society. It helps the person to come into the limelight of the society. There are instances where Trustees even establish themselves in political and other social spheres through this route. Trustees keep on receiving important contacts from other parts of the society through blood donations. Blood camps organizers are usually leaders of the society. Many Trustees uses blood camp organizers for easy networking in personal interest. This networking usually extends to government circles through State Blood Councils and local politicians. Some people start blood banks keeping a larger business plan in mind. Blood banks attached to pathology laboratories want to keep doctors clientage with them. Blood need is one of them and they want to supply to their own group of doctors. Others want to create blood banks as a base to start commercial ventures like plasma fractionation, or as a source for hyperimmune serum and other blood components for business.

Every blood bank starts with a good intention with the highest morals in mind. However, personal interest intrudes into high morals and philanthropy which derail the initial vision and mission of the organization, many a times. There are lots of such interests. One of them is siphoning out money in the garb of NGO status. Other self-interests are to accommodate self or a coterie in the management, expansion of business by opening new blood bank branches, nepotism, sycophancy, putting one professional against another, personal rivalry among trustees etc. Once there is organizational degradation or deviation from the original vision/ mission, technical personnel in the blood banks are affected, especially the medical head. This person becomes sandwiched between moral/ technical standing of the organization and the narrow interest of the Trustees. They are victims of the whims of the Trustees. If you do not have a moral standing and simply follow Trustees’ instructions, you are welcome. In an opposite situation, you are a persona non grata. These types of Trustees look for somebody who complies with legal requirements but is at their mercy. I visited quite a few Trust-run blood banks all over India in my previous job. These are personal observations and inputs given by blood bank workers across the country. One observation was very interesting. Many Trust-run blood banks employ doctors with MBBS (basic medical graduation in the Indian system) degree who are more than 65 years of age. This type of decision takes care of three areas, legal requirement, personal part-time job of a retired person and Trustee can keep absolute control even in technical matters because the head of the technical team is at their mercy. This forces public to brand these type of Trustees as ‘Un-trustful Trustees’.

We have discussed the other side of the story with a few important Trustees all over India. A few of them complained that doctors do not listen to them; they want to run the blood bank (technical part) as they desire. One Trustee said that he had to terminate one doctor because he was mingling with some of his opponents. However, most of the Trustees agreed that lots needs to be done in the NGO sector to bring transparency. They all agreed that Trustees are not above the law and the law of the land should be followed to bring greater transparency in the NGO system. We agree with them that they are offering excellent service to the Indian BTS. If we go by statistics, voluntary blood donation is more in the NGO sector than all other sectors. NGO-run blood banks have a tremendous potential for positive contribution to the system. They have to come out of their selfish motive to earn from the multi billion rupees blood banking industry. One of the major complaints about Trusts is lack of transparency in the management of blood banks. The majority of the Trusts are constituted by main Trustees or promoters. These are usually constituted by family members or a captive population where Trustees can directly influence their decisions.

What is the solution to bring better administration to Trust run blood banks? Trustees should understand that quality of blood supplied to the society is more important than what discounts they are giving on each unit. Trustees are supplying a drug (blood unit) and quality cannot be compromised in the name of charity. Nobody is above the law and everybody has to follow the law of the land, whether it is Drugs and Cosmetics Act and various NACO directives including service charges of blood. It is high time to bring transparency in Trust run blood banks. Let various Trusts have the courage of having broad based Board of Directors (Trustees) of about 12-15 persons so that good constructive criticism an be received for improvement. The tradition of having Trust with family members and own employees should be abandoned. Trust meetings (or Board of Directors) should not be held ‘on paper by circulation’ for long time. Trustees should have the courage to face the reality. One of my friends who is a Trustee has suggested that all Trust should put their audited account on the website (if we have one) and convert the Trust to section 25 company for transparency. However, the last part of the suggestion is not very helpful as per personal observations. Trustees should learn to leave behind narrow personal interest and not to take wrong decision in whims and fancy at the cost of the blood bank. Trustees must respect opinion of technical persons working in blood banks. It is a fact that Trustees/ owners are paying salary to technical staff but without them Trustees/ owners cannot run blood bank activity/ business. Because, blood bank is a medical organization.

We are strongly in favor of continuing services of good NGO-run blood banks and many of them are even standalone blood banks. They have a better number of voluntary donations; they take prompt and proactive actions for blood safety and maintain better quality of components. However, it is important to have some control from the regulatory authority and State Blood Transfusion Councils on the administration of NGO-run blood banks. There should be a wider representation in the Trust (Board of Directors) and it should be constituted by about 15 members and there should not be more than two persons from the same family in the Trust. Please keep one of the technical persons from the blood bank as one of the members of the Trust so that they can express their opinion in the meetings of the Trust/ Board of Directors. Self-interests like nepotism, favoritism, monetary benefits and personal ambitions should be kept aside because NGOs are committed to serve the society. Trustees should follow the rule of self-discipline and let the Blood Bank Officer do his job. Only involve yourself for monitoring of blood bank performance at quarterly or half-yearly intervals or when there is a need to be associated with critical decision-making. Trustees should try to become a ‘referee’ of the game, they need not play the game.

